# Characterization of N-acetyltransferase 1 and 2 polymorphisms and haplotype analysis for inflammatory bowel disease and sporadic colorectal carcinoma

**DOI:** 10.1186/1471-2350-8-28

**Published:** 2007-05-30

**Authors:** Suhal S Mahid, Daniel W Colliver, Nigel PS Crawford, Benjamin D Martini, Mark A Doll, David W Hein, Gary A Cobbs, Robert E Petras, Susan Galandiuk

**Affiliations:** 1 Price Institute of Surgical Research, Department of Surgery, University of Louisville School of Medicine, Louisville, KY 40292, USA; 2 Department of Pharmacology and Toxicology and Brown Cancer Center, University of Louisville School of Medicine, Louisville, KY 40292, USA; 3 Department of Biology, University of Louisville, Louisville, KY 40292, USA; 4 Ameripath, Institute of Gastrointestinal Pathology and Digestive Disease, Oakwood Village, OH 44146, USA

## Abstract

**Background:**

N-acetyltransferase 1 (NAT1) and 2 (NAT2) are polymorphic isoenzymes responsible for the metabolism of numerous drugs and carcinogens. Acetylation catalyzed by NAT1 and NAT2 are important in metabolic activation of arylamines to electrophilic intermediates that initiate carcinogenesis. Inflammatory bowel diseases (IBD) consist of Crohn's disease (CD) and ulcerative colitis (UC), both are associated with increased colorectal cancer (CRC) risk. We hypothesized that *NAT1 *and/or *NAT2 *polymorphisms contribute to the increased cancer evident in IBD.

**Methods:**

A case control study was performed with 729 Caucasian participants, 123 CRC, 201 CD, 167 UC, 15 IBD dysplasia/cancer and 223 controls. *NAT1 *and *NAT2 *genotyping were performed using Taqman based techniques. Eight single nucleotide polymorphisms (SNPs) were characterized for *NAT1 *and 7 SNPs for *NAT2*. Haplotype frequencies were estimated using an Expectation-Maximization (EM) method. Disease groups were compared to a control group for the frequencies at each individual SNP separately. The same groups were compared for the frequencies of *NAT1 *and *NAT2 *haplotypes and deduced NAT2 phenotypes.

**Results:**

No statistically significant differences were found for any comparison. Strong linkage disequilibrium was present among both the *NAT1 *SNPs and the *NAT2 *SNPs.

**Conclusion:**

This study did not demonstrate an association between *NAT1 *and *NAT2 *polymorphisms and IBD or sporadic CRC, although power calculations indicate this study had sufficient sample size to detect differences in frequency as small as 0.05 to 0.15 depending on SNP or haplotype.

## Background

Inflammatory bowel diseases (IBD) are disorders characterized by chronic gastrointestinal inflammation including ulcerative colitis (UC), and Crohn's disease (CD). Both are associated with an increased risk of colorectal cancer (CRC) [1,2]. Colitis associated CRC risk increases with both the duration and extent of the disease [3-6]. The risk of malignant transformation is particularly high for patients having the disease for longer than 8 years, for patients with UC affecting the entire colon [7]. Patients with UC are up to 30-fold more likely to develop CRC and are three times as likely as the general population to die from it [7]. In contrast to sporadic CRC, relatively little is known regarding the pathogenesis of IBD-associated CRC. The continuous state of inflammation and repair in IBD may increase both the frequency and propagation of genetic mutations.

Many enzymes are involved in the metabolism of carcinogens, the present study focused on both N-acetyltransferase (NAT) isoenzymes. NAT1 and NAT2 are both localized to chromosome 8 and are involved in the metabolism of numerous drugs and carcinogens [8,9]. Germline genetic variation within the genes encoding these enzymes can lead to altered phenotypic expression, which in turn impacts on an individual's metabolic capacity. Base pair changes or deletions may affect enzyme stability, expression and/or activity. NAT1 and NAT2 catalyze both N-acetylation and 0-acetylation of arylamine carcinogens [10]. Humans are exposed to arylamine carcinogens such as 4-aminobiphenyl, 2-napthylamine and o-toluidine in cigarette smoke [11].

Conflicting data address *NAT1 *and *NAT2 *polymorphisms and CRC risk. Although *NAT2 *polymorphism is said to modify CRC risk in individuals exposed to heterocyclic amine carcinogens [9], a meta-analysis of 20 case-control studies on NAT2 acetylation status and colon cancer risk reported no consistent effect on CRC risk [12]. To date there has been limited investigation of *NAT *polymorphisms in IBD. The aim of this study was to characterize *NAT1 *and *NAT2 *polymorphisms within a well defined IBD and sporadic CRC population, and to test for association with IBD and/or sporadic CRC.

## Methods

### Control and subject selection

University of Louisville Institutional Review Board approved and written informed consent were obtained from all subjects. Patients were derived from a university colorectal surgery unit. The patient population consisted of 729 Caucasians including 201 unrelated individuals with CD, 167 with UC, 123 with sporadic CRC, 15 with IBD dysplasia or cancer and 223 controls without IBD or CRC. Clinical and demographic information is provided for cases and control in Table 1. Different ethnic groups have varying degrees of susceptibility to inflammatory bowel disease, patients of Jewish ethnicity being more susceptible to IBD and African-Americans less so. The racial/ethnic composition of our patient population comprised 0.9% Asians, 6% African- Americans, 2% Jewish Caucasians and 90% non-Jewish Caucasians. In order to study a homogenous group with an adequate sample size, our study focused upon non-Jewish Caucasians.

**Table 1:** Clinical and demographic characteristics of cases and controls.

**Group**	**Male: Female (n)**	**Average age (yrs)**	**Average age of diagnosis, range(yrs)**	**Family history of IBD (%)**	**Family history of sporadic-CRC (%)**	**Average length of follow up,range(yrs)**
Sporadic-CRC	58:65	62	65 (28–97)	< 2	21	5.2 (0–12)
CD	75:126	46	29(6–81)	38	23	4.6 (0–16)
UC	77:90	48	34(10–75)	25	36	4.3 (0–14)
IBD dysplasia/cancer	6:9	59	45 (12–81)	29	32	4.4 (0–16)
Control	76:147	53	-	9	37	

CRC: colorectal cancer

IBD: inflammatory bowel disease

CD: Crohn's disease

UC: ulcerative colitis

An initial diagnosis of IBD was included, histology in all cases. Following the initial diagnosis, a single specialist gastrointestinal pathologist with a particular interest in IBD reviewed all histology as previous studies have shown that inter-observer variation in can be a significant confounding variable [13-15]. If there was disagreement between the initial diagnosis and that of the specialist pathologist, the latter diagnosis was used.

### Polymorphism detection

Following venipuncture, 10 ml peripheral blood was obtained and Genomic DNA extracted (PURGENE^® ^DNA extraction kit, Gentra Systems Inc., Minneapolis, MN). *NAT1 *and *NAT2 *SNPs; *NAT1*: 97 C > T, 190 C > T, 445G > A, 559C > T, 560G > A, 752A > T, 1088T > A and 1095C > A, and for *NAT2*: 191G> A, 282C > T, 341T > C, 481C > T, 590G > A, 803A > G and 857G > A were determined by TaqMan allele discrimination assays as previously described [16,17]. Amplification reactions were carried out in an ABI Prism 7700 sequence detection system (Applied Biosystems, Foster City, CA). Consensus *NAT1 *and *NAT2 *genotypes were determined as published at . NAT2 phenotypes were deduced from NAT2 genotypes as previously described [18] based on NAT2 phenotypes following recombinant expression of NAT2 alleles [19].

### Statistical analyses

Hardy-Weinberg goodness of fit was determined using an exact test (proc Allele, SAS, Cary, NC). The squared correlation coefficient (r^2^) and Lewontin's standardized disequilibrium coefficient (D'), both measures of linkage disequilibrium (LD), were estimated using SAS proc Allele.

Genotype and Allele frequencies between patients and controls were compared using a R × C contingency table and an exact test.

Haplotype frequencies were estimated from genotype data with unknown phase using Arlequin 2.0 (University of Geneva). The Arlequin program provides exact global p-values for comparison of all haplotypes among all groups as well as exact global p-values for each control group vs. disease comparison for all haplotypes. It also gives the estimated haplotype frequency and its standard error for each haplotype in each group. Tests for differences between control and disease groups for each haplotype frequency were done with a normal distribution (z) test. Within each control-disease group comparison the p-values for each haplotype frequency were then used to compute the false discovery rate p-values (fdrp) [20] using the multi-test procedure of SAS [21]. Only haplotypes with a frequency above 0.15% in at least one group were used in the analysis.

Compounding of type I errors due to multiple testing was controlled by declaring a difference in frequency for a specific haplotype significant only if (1) the global test for homogeneity of all haplotypes in all groups was < 0.05, (2) the global p-value for all haplotypes in a specific control-disease group comparison was < 0.05, (3) the fdrp was < 0.05.

Prospective power for genotype frequency differences was calculated using SAS power RxC macro. The computations involved setting a single genotype in a single group to be different by the amount delta from its frequency in the control and all other groups except the IBD dysplasia/cancer group, which was too small to conduct meaningful power calculations. Power was then computed for a range of different values of delta. Approximate prospective power of the haplotype analysis was computed by simulation of sampling from a control population and disease population in which a particular haplotype (focal haplotype) frequency in the disease group was set to be greater than the frequency in the control group by amount delta. All other haplotypes in the disease group were adjusted proportionally to make the sum of all haplotype frequencies equal one. Each sample was tested for a difference between the control and disease group using Proc haplotype (SAS), each value of delta, 10,000 simulations were performed and power calculated as the proportion of tests in which the null hypothesis was rejected. The null hypothesis was the frequency of the focal haplotype is the same in the two groups. To account for multiple testing when comparing 3 groups, the Bonferroni method was used giving a significance level of α/κ = 0.0167 for each test.

## Results

All 729 participants were genotyped for *NAT1 *SNPs. Four subjects who could not be genotyped for *NAT2 *were excluded from analysis. Twenty different genotypes were observed for *NAT1 *(Table 2), 26 genotypes were found for *NAT2 *(Table 3). Thirteen *NAT2 *genotypes are associated with slow acetylation phenotype, 10 genotypes with "intermediate" acetylation phenotype and 3 with rapid acetylation phenotype.

**Table 2:** *NAT1 *genotype frequency.

***NAT1 *Genotype**	**Sporadic-CRC**		**IBD benign**		**IBD dysplasia/cancer**		**Control**	
	No.	%	No.	%	No.	%	No.	%
NAT1*4/*4	73	59.35	205	55.7	9	60	131	58.75
NAT1*4/*10	28	22.77	104	28.3	6	40	61	27.35
NAT1*10/*10	9	7.32	18	4.89			9	4.03
NAT1*4/*14A or *10/*14B	4	3.25	13	3.53			7	3.14
NAT1*4/*11B	0	0	6	1.63			5	2.24
NAT1*4/*14B	2	1.63	3	0.81			2	0.9
NAT1*10/*11B	1	0.81	2	0.54			2	0.9
NAT1*4/*3	2	1.63	3	0.81			1	0.45
NAT1*4/*15	0	0	1	0.27			1	0.45
NAT1*4/*11A or *3/*11B	1	0.81	0	0			1	0.45
NAT1*3/*11A	0	0	0	0			1	0.45
NAT1*10/*15	0	0	0	0			1	0.45
NAT1*4/*17	0	0	6	1.63			1	0.44
NAT1*3/*3	1	0.81	2	0.54			0	0
NAT1*10/*17	0	0	2	0.54			0	0
NAT1*10/*22	1	0.81	1	0.27			0	0
NAT1*4/*22	0	0	1	0.27			0	0
NAT1*3/*14A	1	0.81	0	0			0	0
NAT14A/*22	0	0	1	0.27			0	0
Total	123		368		15		223	

Genotypes listed by order of decreasing frequency in control group

IBD: inflammatory bowel disease

CRC: colorectal cancer

**Table 3:** *NAT2 *genotype frequency.

***NAT2 *Genotype**	**Sporadic-CRC**		**IBD benign**		**IBD dysplasia/cancer**		**Controls**		**Phenotype**
	No.	%	No.	%	No.	%	No.	%	
NAT2*5B/*6A	26	21.3	85	23.22	5	33.33	54	24.3	Slow
NAT2*4/*5B	22	18	72	19.67	1	6.66	39	17.5	Intermediate
NAT2*5B/*5B	17	13.9	54	14.75	3	20	32	14.4	Slow
NAT2*4/*6A	24	19.7	48	13.11	4	26.66	30	13.5	Intermediate
NAT2*6A/*6A	16	13.2	20	5.46			21	9.45	Slow
NAT2*4/*4	3	2.45	26	7.1			16	7.2	Rapid
NAT2*6A/*7B	3	2.45	6	1.63			3	1.36	Slow
NAT2*5B/*7B	2	1.63	8	2.18			5	2.25	Slow
NAT2*5B/*5C	2	1.63	2	0.54			4	1.8	Slow
NAT2*5A/*6A	0	0	2	0.54			2	0.9	Slow
NAT2*5B/*13	0	0	5	1.36	1	6.66	2	0.9	Intermediate
NAT2*4/*5A	0	0	5	1.36			2	0.9	Intermediate
NAT2*4/*7B	2	1.63	5	1.36	1	6.66	2	0.9	Intermediate
NAT2*5C/*6A	0	0	3	0.819			2	0.9	Slow
NAT2*5A/*5B	1	0.81	8	2.18			1	0.45	Slow
NAT2*5A/*5C	0	0	2	0.54			1	0.45	Slow
NAT2*5B/*12A	1	0.81	0	0			1	0.45	Intermediate
NAT2*5C/*5C	0	0	1	0.27			1	0.45	Slow
NAT2*4/*12A	0	0	0	0			1	0.45	Rapid
NAT2*4/*5C	1	0.81	7	1.91			1	0.45	Intermediate
NAT2*6A/*12A	0	0	0	0			1	0.45	Intermediate
NAT2*6A/*13	1	0.81	1	0.27			1	0.45	Intermediate
NAT2*4/*13	0	0	2	0.54			0	0	Rapid
NAT2*5A/*13	0	0	1	0.27			0	0	Intermediate
NAT2*5A/*5A	1	0.81	1	0.27			0	0	Slow
NAT2*5C/*7B	0	0	2	0.54			0	0	Slow
Total	122		366		15		222		

Genotypes listed by order of decreasing frequency in control group

IBD: inflammatory bowel disease

CRC: colorectal cancer

### *NAT1*

None of the eight SNPs showed any significant departure from Hardy-Weinberg equilibrium proportions in any of the groups. Significant pairwise linkage disequilibrium (LD) was found between three of the 8 SNPs; C1095A, G560A and T1088A.

### Power of *NAT1 *analyses

Two SNPs (G560A and C1095A) were selected for power analysis, G560A had very low levels of polymorphism (genotype frequencies; GG: 0.954, GA: 0.046). C1095A was more polymorphic (genotype frequencies; AA: 0.056; CA: 0.332; CC: 0.612).

The groups in the analysis were CRC, CD, control, and UC with sample sizes 123, 201, 223, and 167, respectively. UC was the focal group for all analyses and GA the focal genotype for the G560A SNP analyses. AA and CA were the focal genotypes for C1095A analyses. The power against delta = 0.1 for G560A was 0.95, so the power against a difference of = 0.1 was good, but fell of rapidly for smaller delta values. The power of the focal genotype AA for C1095A, for delta = 0.1 was 0.843, while the power for CA the focal genotype for C1095A for delta = 0.1 was 0.384. Examination of the power curve indicates good power against a delta ≥ 0.125 for the AA genotype and for the CA genotype good power against a delta ≥ 0.175.

The haplotype identifier number, SNP composition and frequency in the control group for the four focal haplotypes were as follows: AACCCAGA, < 0.001; AACTCGGA, 0.001; AACCCGGA, 0.183 and ACCCCGGT, 0.766. The first and second of these haplotypes were low frequency haplotypes and the third and fourth were high frequency haplotypes. Power curves were substantially different for different haplotypes. Examination of the power curves indicated power for against a change of delta = 0.10 to be 0.999, 0.046, 0.641, and 0.632, respectively, for the above 4 haplotypes. For delta = 0.15 the powers were 1.000, 0.057, 0.958 and 0.057 respectively.

### *NAT1 *case-control association studies

*NAT1 *genotypes are shown in Table 1. There were no significant differences among any groups with respect to genotype or allele frequencies. The Cochran-Armitage test gave no significant differences for genotype frequencies between sporadic-CRC, IBD, and control populations.

The most common allele was NAT1*4, in agreement with published literature [9]. The global null hypothesis of no difference in haplotype frequencies between groups was not rejected and there were no differences in haplotype frequencies among any groups when pairwise testing was performed (p = 0.851).

### *NAT2*

None of the seven *NAT2 *SNPs showed any significant departure from Hardy-Weinberg equilibrium proportions in any of the groups. Almost all pairwise combinations of the 7 SNPs showed significant LD, except G857A which had highly significant LD with C282T and T341C only.

### Power of *NAT2 *analyses

Two SNPs (C282T and G857A) were selected for power analysis. The G857A SNP was not very polymorphic (genotype frequencies, GG: 0.948, GA: 0.052). The C282T SNP was more polymorphic (genotype frequencies, CC: 0.458, CT: 0.44, TT: 0.102). The groups and sample sizes in the analysis were CRC (n = 123), CD (n = 201), control (n = 223), and UC (n = 167). UC was the focal group for all analyses and GA the focal genotype for the G857A SNP analysis and TT and CT the focal genotypes for the C282T analysis. For G857A, the power against delta= 0.1 was 0.963. The power against a difference ≥ 0.1 was good, but fell off rapidly for smaller delta values. For C282T, the TT focal genotype yielded a power of 0.723 for delta = 0.1, whereas the CT focal genotype yielded a power of 0.346 for delta = 0.1. Examination of the power curve indicated that there was good power against a delta ≥ 0.15 for the TT genotype and there was good power against a delta ≥ 0.2 for the CT genotype.

The haplotype identification number, SNP composition, and frequency in the control group for the four focal haplotypes were as follows: ACCGGGC, 0.002; ATCGAGT, 0.307; ATCGGGT, 0.007 and GCTGGGC, 0.383. The power curves indicated more power for detecting an increase in frequency for some haplotypes than for others. The power against a delta = 0.10 for the above four haplotypes are 0.284, 0.555, 0.127 and 0.317 respectively. For delta = 0.15 the powers were 0.689, 0.887, 0.288, and 0.766 respectively.

### *NAT2 *case-control association studies

All 7 *NAT2 *SNPs were identified in our population. NAT2 *5B was the most common allele in agreement with previous studies in Caucasians [22]. There was no difference among groups with respect to single SNP analysis using the Cochran-Armitage test, homogeneity of genotype frequencies or allele frequencies. There was no difference in haplotype frequencies among groups (p = 0.468) and no pairwise differences.

There was no correlation between disease group and *NAT2 *acetylation phenotype [Global p-value = 0.354, χ^2 ^= 8.62 (6 d.f)].

## Discussions and conclusion

NAT1 and NAT2 activity have been described in human intestine; both are involved in the metabolism of arylamine carcinogens such as 4-aminobiphenyl found in tobacco smoke [11,23]. The primary step in hepatic 4-aminobiphenyl metabolism involves two competing pathways: N-acetylation and N-oxidation (hydroxylation). Individuals who exhibit slow acetylator phenotypes produce higher concentrations of 4-aminobiphenyl derived hemoglobin adducts and have a higher risk of smoking related cancers [11,24]. Within smokers, arylamine carcinogen levels are greater as a consequence of the slow N-acetylator phenotype characterized by homozygosity for less active variant alleles. Differences in *NAT1 *or *NAT2 *haplotypes with respect to acetylator phenotypes could be considered as contributing factors in increasing exposure to carcinogenic products.

The role of *N*-acetyltransferases in cancer predisposition varies between different organs as might be expected with tissue-specific expression of *NAT1 *and *NAT2 *[8]. Some early studies suggested an increased CRC risk associated with the rapid acetylation phenotype [22,24,25]. However, the increased risk was relatively small and much progress has been made in the accurate determination of both genotype and acetylation status. These associations are strongest with documented exposure to heterocyclic amine carcinogens in the diet [22]. More recently, a study on 275 patients with colon cancer and 343 controls revealed a significant association between the slow acetylation genotype and early age of onset [26]. Some previous studies have reported associations between the NAT1*10 allele and CRC, particularly in association with rapid NAT2 acetylators, while others have reported no association [9]. These contradictory results may be due to the small number of CRC cases examined; however, the role of NAT2 acetylation in CRC risk still remains unclear. The role of *NAT1 *and *NAT2 *polymorphisms and their association with IBD is even less clear. Few papers address *NAT *polymorphisms in association with IBD [27,28]. The true risk of patients with IBD developing CRC is debated. It is thought by some to increase by 0.5 – 1.0% yearly beginning 8 – 10 years after diagnosis [29,30]. However, many of our patients have surgical intervention due to disease activity and accurate prediction of cancer risk in IBD patients is confounded by whether or not they have had surgical intervention [2,31].

In our overall population of 281 UC patients and 460 CD patients, 18 UC patients (6.4%) and 15 CD patients (3.2%) had a confirmed diagnosis of dysplasia or cancer. For UC, nine of these 18 patients had colorectal cancer and nine had dysplasia. For CD, seven of these 15 patients had colorectal cancer and eight had dysplasia. However, genomic DNA for analysis was not available for all these patients.

We examined *NAT1 and NAT2 *polymorphisms in genomic DNA and found no association between *NAT1 *and *NAT2 *polymorphisms with either benign IBD, IBD dysplasia/cancer or sporadic colorectal cancer, compared to controls. Power calculations for both single marker and haplotype analyses for *NAT1 *and *NAT2*, indicated that differences in frequency between control and disease groups of 0.05 and higher had a very high chance (80 – 90%) of detection. The greatest power of our study was in detecting genotypes or haplotypes that are rare in the control group and increased in frequency in disease groups. For many haplotypes, this study had a good chance of detecting differences as small as 0.15. Thus, if a strong relationship between any of the three conditions and NAT1 or NAT2 SNPs existed, they would likely have been found by this study.

A recent report from Japan suggested an association of the NAT2*7B haplotype (p = 0.013) with CD in a cohort of 60 CD, 95 UC and 200 gender matched unrelated controls [32]. Our inability to replicate this finding may be due to a number of factors including the presence of heterogeneity between populations of different race, differences in LD or population differences in allele frequencies of interacting genes. Different environmental exposures may also have an effect on these diseases.

Strengths of this study are the relatively large sample size, reproducible methods and stringent study design. Since this study was restricted to Caucasians, further studies across different ethnic groups are needed. Future studies that consider age of onset also are needed.

## Competing interests

The author(s) declare that they have no competing interests.

## Authors' contributions

SSM performed genotyping, drafted the manuscript and responsible for coordination of laboratory work; authors DWC, NPSC, BDM and MAD performed genotyping and data interpretation; GAC performed statistical analyses; REP performed re-review of IBD histology; DWH and SG collected all patient samples, participated in the design of the study, coordination, and manuscript preparation and obtained funding for this work. All authors read and approved the final manuscript.

## Pre-publication history

The pre-publication history for this paper can be accessed here:


